# The Unfinished Journey After Major Surgery: Interhospital Transfer and Care Fragmentation

**DOI:** 10.1002/wjs.70502

**Published:** 2026-07-14

**Authors:** Rida Ejaz, Qaidar Alizai, Lorenza Arena, Kizuki Yuza, Charalampos M. Charalampous, Abdulaziz Elemosho, Timothy M. Pawlik

**Affiliations:** ^1^ Department of Surgery The Ohio State University Wexner Medical Center and James Comprehensive Cancer Center Columbus Ohio USA

**Keywords:** care fragmentation, continuity of Patient care, operative, patient readmission, patient transfer, surgical procedures, treatment outcome

## Abstract

**Background:**

Interhospital transfer (IHTR) is commonly used to access specialized surgical care, yet post‐discharge readmission destination among transferred surgical patients remains poorly characterized. Therefore, the association of IHTR and hospital transfer burden with care fragmentation after major surgery was evaluated.

**Methods:**

Adults aged ≥ 18 years undergoing major surgery between 2016 and 2020 were identified from the Nationwide Readmissions Database. Procedures included coronary artery bypass grafting, abdominal aortic aneurysm repair, esophagectomy, hepatectomy, rectal resection, pneumonectomy, pancreatectomy, and colectomy. Patients were stratified as IHTR versus non‐transfer (NTR). Care fragmentation was defined as 30‐day readmission to a hospital different from the index surgical hospital. Multivariable regression evaluated the association of IHTR with postoperative and readmission outcomes. Hospital‐level analyses evaluated the association between hospital transfer burden and care fragmentation.

**Results:**

Among 1,831,450 patients, 77,498 (4.2%) were categorized as IHTR and 1,753,952 (95.8%) as NTR. Compared with NTR patients, IHTR patients had higher comorbidity burden (median Elixhauser groups, 6 [IQR, 4–8] vs. 4 [IQR, 2–5]) and more commonly underwent nonelective surgery (*n* = 67,184 [86.7%] vs. *n* = 688,802 [39.3%]; both *p* < 0.001). Thirty‐day readmission was modestly higher among IHTR versus NTR patients (*n* = 12,743 [16.4%] vs. *n* = 211,311 [12.1%]; adjusted odds ratio [aOR], 1.02; 95% CI, 1.00–1.05), whereas care fragmentation among readmitted patients was substantially more common after IHTR (*n* = 6424 [50.4%] vs. *n* = 40,327 [19.1%]; aOR = 3.75; 95% CI, 3.58–3.92). This association was consistent among nonelective (aOR = 4.05; 95% CI, 3.86–4.26) and elective patients (aOR = 3.46; 95% CI, 3.10–3.86). Greater hospital transfer burden was associated with higher care fragmentation odds overall and among NTR readmitted patients (both aOR, 1.08).

**Conclusion:**

IHTR was strongly associated with care fragmentation after major surgery, despite a modest association with 30‐day readmission overall. Greater hospital transfer burden was also associated with hospital‐level care fragmentation, suggesting that transfer status and hospital transfer burden may help identify patients and hospitals at increased risk for fragmented post‐discharge acute care within regionalized surgical systems.

## Graphical abstract

Interhospital transfer (IHTR) is commonly used to access specialized surgical care, yet post‐discharge readmission destination among transferred surgical patients remains poorly characterized. Therefore, the association of IHTR and hospital transfer burden with care fragmentation after major surgery was evaluated. IHTR was strongly associated with care fragmentation after major surgery, despite a modest association with 30‐day readmission overall. Greater hospital transfer burden was also associated with hospital‐level care fragmentation, suggesting that transfer status and hospital transfer burden may help identify patients and hospitals at increased risk for fragmented post‐discharge acute care within regionalized surgical systems.

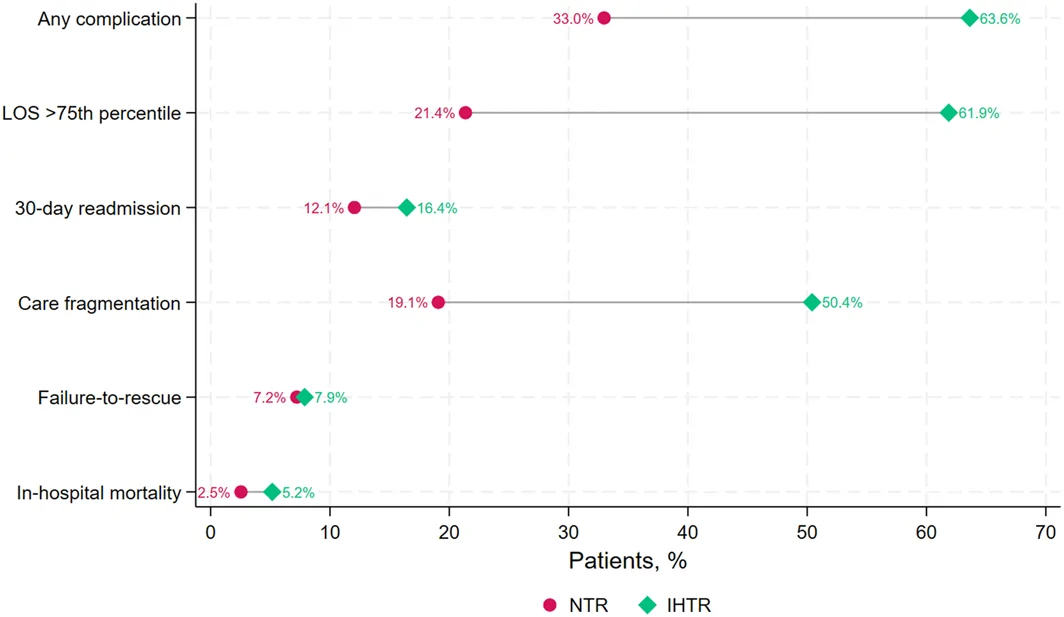

## 1 Introduction

Interhospital transfer (IHTR) is a common feature of regionalized surgical care, allowing patients to access services, procedural expertise, and hospital resources that may not be available at the originating facility. Approximately 1.5 million patients are transferred between acute care hospitals each year in the United States, while transfer rates among surgical populations have been reported to range from 3% to 7% of inpatient procedures [1, 2, 3]. Transferred surgical patients often have higher acuity, greater comorbidity burden, and higher resource utilization than non‐transferred patients [4, 5, 6]. As such, IHTR identifies a distinct subgroup of major surgical patients whose care begins across more than one institution.

Care fragmentation, defined as readmission to a hospital different from the index surgical hospital, is an important but incompletely characterized post‐discharge outcome after major surgery [7, 8]. Among Medicare beneficiaries, approximately 1 in 4 older patients readmitted after major surgery were readmitted to a non‐index hospital, and systematic review data suggest that non‐index readmissions account for 10%–47% of 30‐day surgical readmissions [7, 8]. Prior work has largely examined either IHTR during the index hospitalization or care fragmentation after discharge as separate phenomena. Studies of IHTR have focused mainly on index‐admission outcomes such as morbidity, mortality, resource utilization, and failure‐to‐rescue, whereas studies of care fragmentation have generally not focused on patients transferred into the surgical hospitalization [9, 10, 11, 12]. Therefore, whether IHTR is associated with the location of subsequent readmission remains poorly characterized.

The current study sought to address this gap by characterizing the association between IHTR and care fragmentation after major surgery using a national cohort. In addition, because hospitals vary in the proportion of surgical patients admitted via transfer, hospital transfer burden was evaluated in relation to hospital‐level care fragmentation, including among patients who were not transferred into the index hospitalization [13]. We hypothesized that IHTR would be associated with higher odds of care fragmentation among readmitted patients and that greater hospital transfer burden would be associated with higher hospital‐level care fragmentation.

## 2 Methods

### 2.1 Data Source and Study Population

Adult patients who were hospitalized for a major surgical procedure between 2016 and 2020 were identified from the Nationwide Readmissions Database (NRD). The NRD is an all‐payer readmission database maintained by the Healthcare Cost and Utilization Project and allows tracking of same‐state readmissions within a calendar year [14]. Major surgery was defined as coronary artery bypass grafting (CABG), abdominal aortic aneurysm (AAA) repair, esophagectomy, hepatectomy, rectal resection, pneumonectomy, pancreatectomy, and colectomy using International Classification of Diseases, Tenth Revision (ICD‐10) procedure codes. These procedures have been used in prior claims‐based surgical outcomes research [15, 16].

Patients aged < 18 years, without an eligible procedure, or with missing data required to define transfer status or readmission outcomes were excluded. For patients with > 1 eligible surgical hospitalization during the study period, only the first eligible operation was retained as the index admission. Due to the retrospective design and use of deidentified NRD data, the study was deemed exempt from institutional review board review, and informed consent was waived. The study followed the Strengthening the Reporting of Observational Studies in Epidemiology (STROBE) reporting guidelines.

### 2.2 Exposure

The primary exposure was interhospital transfer (IHTR) into the index surgical hospitalization, identified using the NRD same‐day event variable. IHTR was defined as transfer involving two discharges from different hospitals, same‐day stays involving two discharges from different hospitals, or same‐day stays involving three or more discharges at the same or different hospitals. All other patients were categorized into the non‐transfer (NTR) group. Transfer out during the index hospitalization was not classified as care fragmentation unless followed by a qualifying 30‐day readmission to a hospital different from the index surgical hospital. Study definitions and eligible transfer‐readmission pathways are shown in Figure S1.

For hospital‐level analyses, hospital transfer burden was defined as the proportion of included major surgical patients at each index hospital admitted via IHTR. This study‐defined measure characterized transfer intensity within each hospital's surgical population, consistent with prior work using transfer frequency, hospital procedural volume, and interhospital transfer‐network patterns as markers of hospital capability, institutional experience, and regionalized care pathways [15, 17, 18, 19]. Hospital transfer burden was modeled continuously per 1‐percentage‐point increase.

### 2.3 Covariates

Patient‐level covariates included age, sex, insurance status, ZIP‐code income quartile, weekend admission, surgical acuity, surgery type, and comorbidity burden. Insurance status was categorized as Medicare, Medicaid, private insurance, self‐pay, no charge, or other. Surgical acuity was categorized as elective versus nonelective surgery using the administrative elective surgery indicator. Comorbidity burden was assessed using the total number of Elixhauser comorbidity groups [14, 20].

Hospital‐level covariates included hospital bed size, hospital ownership, hospital urban/teaching status, metropolitan location, and hospital operative volume. Hospital bed size was categorized as small, medium, or large using HCUP hospital bed‐size categories, which account for hospital region, location, and teaching status. Hospital ownership was categorized as government non‐federal, private not‐for‐profit, or private investor‐owned. Hospital urban/teaching status was categorized as rural, urban nonteaching, or urban teaching. Hospital operative volume was defined as the total number of included major surgical procedures performed at each hospital during the study period. Log‐transformed hospital operative volume was used in hospital‐level models.

### 2.4 Outcomes of Interest

The primary outcome was care fragmentation among patients readmitted within 30 days of discharge from the index surgical hospitalization. Care fragmentation was defined as readmission to a hospital different from the index surgical hospital, determined by comparing the deidentified NRD hospital identifier from the readmission record with the hospital identifier from the index admission [7, 8, 10]. Patients who were discharged alive after the index surgical hospitalization and did not experience a 30‐day readmission were outside the analytic denominator for care fragmentation. Patients readmitted to the same hospital were categorized as non‐fragmented, whereas patients readmitted to a different hospital were categorized as fragmented. A secondary patient‐level outcome was any 30‐day readmission in the full surgical cohort.

Secondary outcomes included index‐admission complications, index length of stay (LOS), prolonged LOS, discharge home, failure‐to‐rescue (FTR), and in‐hospital mortality. FTR was defined as death following a documented complication [21, 22]. Prolonged LOS was defined as LOS greater than the 75th percentile of the cohort distribution.

### 2.5 Statistical Analyses

Categorical variables were reported as frequencies and proportions and compared using χ^2^ tests. Continuous variables were reported as medians with interquartile ranges and compared using Wilcoxon rank‐sum tests. Baseline characteristics and outcomes were compared between IHTR and NTR patients.

Missingness assessment and handling are summarized in Supporting Information S1: Table 1. Care fragmentation was evaluated only among patients with 30‐day readmission; patients without readmission were outside the analytic denominator and were not considered missing for this outcome.

Multivariable logistic regression was used to evaluate the association between IHTR and care fragmentation among readmitted patients. Models adjusted for age, sex, insurance status, ZIP income quartile, weekend admission, surgical acuity, surgery type, Elixhauser comorbidity burden, hospital bed size, hospital ownership, hospital urban/teaching status, metropolitan location, and hospital operative volume. Standard errors were clustered at the hospital level. A separate model evaluated the association between IHTR and 30‐day readmission in the full cohort. Analyses were repeated after stratification by surgical acuity. Results were reported as adjusted odds ratios (aORs) with 95% confidence intervals (CIs).

The primary hospital‐level model used a generalized linear model with binomial family and logit link. The numerator was fragmented readmissions, and the denominator was total 30‐day readmissions from each hospital. Models adjusted for log‐transformed hospital operative volume, hospital bed size, hospital ownership, hospital urban/teaching status, and metropolitan location. A secondary hospital‐level model restricted the numerator and denominator to NTR patients only.

Sensitivity analyses included adjustment for patient resident status, restriction to in‐state residents, and additional adjustment for index‐admission complications, LOS, and discharge disposition. Propensity‐based sensitivity analyses included truncated inverse probability of treatment weighting, doubly adjusted inverse probability weighting, and 1:1 propensity‐score matching among readmitted patients. Hospital‐level linear rate models were repeated among hospitals with ≥ 10, ≥ 20, and ≥ 30 readmissions. Additional supplemental analyses evaluated procedure‐specific care fragmentation, temporal trends, sex‐based associations, and readmission‐course outcomes by readmission destination.

All analyses were conducted using Stata, version 16.1. Statistical tests were 2‐sided, with *p* < 0.05 considered statistically significant.

## 3 Results

### 3.1 Baseline Characteristics

A total of 1,831,450 adult patients undergoing a major surgical procedure were included in the analytic cohort. Among them, 1,753,952 (95.8%) were categorized as non‐transfer (NTR), while 77,498 (4.2%) were categorized as interhospital transfer (IHTR). Median age was similar between groups (68 [IQR, 58–76] years among NTR vs. 68 [IQR, 59–76] years among IHTR); however, IHTR patients were more often male (65.8%, *n* = 51,012 vs. 57.2%, *n* = 1,004,008) and had a higher comorbidity burden (median Elixhauser groups: 6 [IQR, 4–8] vs. 4 [IQR, 2–5]; both *p* < 0.001) (Table 1).

**TABLE 1:** Baseline characteristics comparison of the non‐transfer and interhospital transfer groups.

Characteristic	Total (*N* = 1,831,450)	NTR (*N* = 1,753,952)	IHTR (*N* = 77,498)	*p* value
Age, years, median (IQR)	68 (58–76)	68 (58–76)	68 (59–76)	< 0.001
Sex				< 0.001
Male	1,055,020 (57.61)	1,004,008 (57.24)	51,012 (65.82)	
Female	776,430 (42.39)	749,944 (42.76)	26,486 (34.18)	
Insurance status				< 0.001
Medicare	1,080,658 (59.01)	1,032,793 (58.88)	47,865 (61.76)	
Medicaid	143,866 (7.86)	136,701 (7.79)	7165 (9.25)	
Private	522,326 (28.52)	504,226 (28.75)	18,100 (23.36)	
Self‐pay	34,480 (1.88)	32,315 (1.84)	2165 (2.79)	
No charge	5364 (0.29)	4986 (0.28)	378 (0.49)	
Other	44,756 (2.44)	42,931 (2.45)	1825 (2.35)	
ZIP income quartile				< 0.001
Q1, lowest	449,239 (24.53)	429,517 (24.49)	19,722 (25.45)	
Q2	492,424 (26.89)	471,060 (26.86)	21,364 (27.57)	
Q3	474,603 (25.91)	454,746 (25.93)	19,857 (25.62)	
Q4, highest	415,184 (22.67)	398,629 (22.73)	16,555 (21.36)	
Weekend admission	179,124 (9.78)	161,810 (9.23)	17,314 (22.34)	< 0.001
Nonelective surgery	755,986 (41.28)	688,802 (39.27)	67,184 (86.69)	< 0.001
Elective surgery	1,075,464 (58.72)	1,065,150 (60.73)	10,314 (13.31)	
Surgery type				< 0.001
AAA	272,549 (14.88)	265,069 (15.11)	7480 (9.65)	
Esophagectomy	15,939 (0.87)	15,607 (0.89)	332 (0.43)	
Hepatectomy	33,309 (1.82)	32,876 (1.87)	433 (0.56)	
Rectal resection	131,939 (7.20)	129,618 (7.39)	2321 (2.99)	
CABG	487,214 (26.60)	435,998 (24.86)	51,216 (66.09)	
Colectomy	673,183 (36.76)	661,689 (37.73)	11,494 (14.83)	
Pancreatectomy	41,493 (2.27)	40,424 (2.30)	1069 (1.38)	
Pneumonectomy	175,824 (9.60)	172,671 (9.84)	3153 (4.07)	
Elixhauser comorbidity burden, median (IQR)	4 (2–6)	4 (2–5)	6 (4–8)	< 0.001
Hospital bed size				< 0.001
Small	206,567 (11.28)	199,770 (11.39)	6797 (8.77)	
Medium	463,083 (25.29)	445,434 (25.40)	17,649 (22.77)	
Large	1,161,800 (63.44)	1,108,748 (63.21)	53,052 (68.46)	
Hospital ownership				< 0.001
Government, non‐federal	180,544 (9.86)	172,709 (9.85)	7835 (10.11)	
Private, not‐for‐profit	1,435,782 (78.40)	1,376,497 (78.48)	59,285 (76.50)	
Private, investor‐owned	215,124 (11.75)	204,746 (11.67)	10,378 (13.39)	
Hospital teaching status				< 0.001
Rural	331,762 (18.11)	321,448 (18.33)	10,314 (13.31)	
Urban nonteaching	1,431,876 (78.18)	1,366,659 (77.92)	65,217 (84.15)	
Urban teaching	67,812 (3.70)	65,845 (3.75)	1967 (2.54)	
Metropolitan hospital	1,544,695 (84.34)	1,481,887 (84.49)	62,808 (81.04)	< 0.001
Hospital operative volume, median (IQR)	172 (84–306)	167 (81–300)	266 (138–424)	< 0.001

*Note:* Values are reported as frequency (percentage) unless mentioned otherwise. Continuous variables are presented as median (interquartile range). *p*‐values were derived from χ^2^ tests for categorical variables and Wilcoxon rank‐sum tests for continuous variables.

Abbreviations: AAA, abdominal aortic aneurysm; CABG, coronary artery bypass grafting; IHTR, interhospital transfer; IQR, interquartile range; NTR, non‐transfer; ZIP, Zone Improvement Plan.

IHTR patients were more likely to have undergone nonelective surgery (86.7%, *n* = 67,184 vs. 39.3%, *n* = 688,802) and weekend admission (22.3%, *n* = 17,314 vs. 9.2%, *n* = 161,810; both *p* < 0.001). Procedure type differed between groups, with CABG more common among IHTR patients (66.1% vs. 24.9%) and colectomy more common among NTR patients (37.7% vs. 14.8%; *p* < 0.001). IHTR patients were more often treated at large hospitals and hospitals with higher operative volume (median 266 [IQR, 138–424] vs. 167 [IQR, 81–300]; both *p* < 0.001) (Table 1).

### 3.2 Readmission and Care Fragmentation by Transfer Status

**FIGURE 1:**
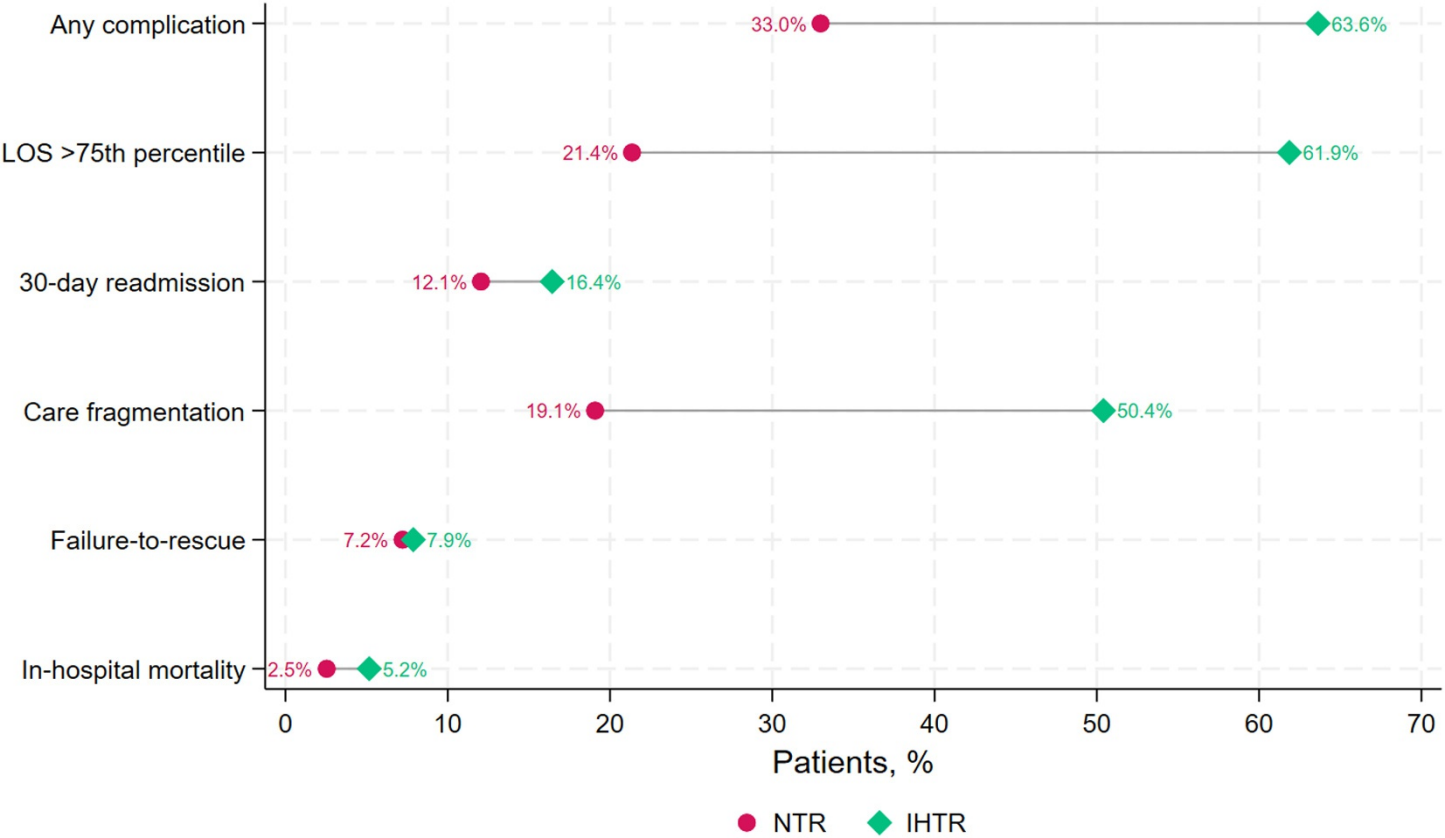
Outcome rates by interhospital transfer status. Dumbbell plot comparing unadjusted outcome rates among patients categorized as interhospital transfer (IHTR) versus non‐transfer (NTR). Care fragmentation was evaluated among patients with 30‐day readmission and was defined as readmission to a hospital different from the index surgical hospital. Failure‐to‐rescue was evaluated among patients with a documented complication. IHTR, interhospital transfer; LOS, length of stay; NTR, non‐transfer.

Compared with NTR patients, IHTR patients had higher unadjusted rates of any complication (63.6% vs. 33.0%), longer median index length of stay (LOS) (14 [IQR, 10–22] vs. 6 [IQR, 3–9] days), and higher prolonged LOS (61.9% vs. 21.4%; all *p* < 0.001). Discharge home was less common among IHTR patients (31.3% vs. 57.7%; *p* < 0.001) (Table 2; Figure 1).

**TABLE 2:** Univariable comparison of outcomes between non‐transfer and interhospital transfer groups.

Outcome	Total (*N* = 1,831,450)	NTR (*N* = 1,753,952)	IHTR (*N* = 77,498)	*p* value
Any complication	627,828 (34.28)	578,518 (32.98)	49,310 (63.63)	< 0.001
Index LOS, days, median (IQR)	6 (3–10)	6 (3–9)	14 (10–22)	< 0.001
LOS > 75th percentile	422,551 (23.07)	374,600 (21.36)	47,951 (61.87)	< 0.001
Discharge home	1,035,393 (56.53)	1,011,136 (57.65)	24,257 (31.30)	< 0.001
30‐day readmission	224,054 (12.23)	211,311 (12.05)	12,743 (16.44)	< 0.001
Care fragmentation^a^	46,751 (20.87)	40,327 (19.08)	6424 (50.41)	< 0. 001
Failure‐to‐rescue^b^	45,652 (7.27)	41,766 (7.22)	3886 (7.88)	< 0.001
In‐hospital mortality	48,598 (2.65)	44,598 (2.54)	4000 (5.16)	< 0.001

*Note:* Values are reported as frequency (percentage) unless mentioned otherwise. Continuous variables are presented as median (interquartile range). *p*‐values were derived from χ^2^ tests for categorical variables and Wilcoxon rank‐sum tests for continuous variables.

Abbreviations: IHTR, interhospital transfer; IQR, interquartile range; LOS, length of stay; NTR, non‐transfer.

^a^ Analytic cohort for care fragmentation included patients with 30‐day readmission: total *n* = 224,054; NTR *n* = 211,311; IHTR *n* = 12,743. Care fragmentation was defined as readmission to a hospital different from the index surgical hospital.

^b^ Analytic cohort for failure‐to‐rescue included patients with a documented complication: total *n* = 627,828; NTR *n* = 578,518; IHTR *n* = 49,310.

Thirty‐day readmission occurred among 224,054 (12.2%) patients overall, including 211,311 (12.1%) NTR patients and 12,743 (16.4%) IHTR patients (*p* < 0.001). Among patients with 30‐day readmission, care fragmentation occurred among 46,751 (20.9%) patients. Care fragmentation was substantially more common among IHTR versus NTR patients (50.4%, *n* = 6424 vs. 19.1%, *n* = 40,327; *p* < 0.001) (Table 2; Figure 1). Among patients with a documented complication, failure‐to‐rescue was 7.9% (*n* = 3886) among IHTR patients versus 7.2% (*n* = 41,766) among NTR patients (*p* < 0.001). In‐hospital mortality was also higher among IHTR patients (5.2%, *n* = 4000 vs. 2.5%, *n* = 44,598; *p* < 0.001) (Table 2; Figure 1).

### 3.3 Adjusted Patient‐Level Analyses

On multivariable analysis, IHTR was associated with higher odds of any complication (adjusted odds ratio [aOR], 1.81; 95% CI, 1.76–1.86; *p* < 0.001), prolonged LOS greater than the 75th percentile (aOR, 4.07; 95% CI, 3.94–4.20; *p* < 0.001), and lower odds of discharge home (aOR, 0.76; 95% CI, 0.73–0.79; *p* < 0.001). IHTR was associated with longer index LOS (incidence rate ratio [IRR], 1.73; 95% CI, 1.70–1.75; *p* < 0.001) (Table 3).

**TABLE 3:** Multivariable regression analyses for the association of interhospital transfer with postoperative and readmission outcomes, with reference to the non‐transfer group.

Outcome	Adjusted OR/IRR (95% CI)	*p* value
Any complication^a^	1.81 (1.76–1.86)	< 0.001
Index LOS, days^b^	1.73 (1.70–1.75)	< 0.001
LOS > 75th percentile^a^	4.07 (3.94–4.20)	< 0.001
Discharge home^a^	0.76 (0.73–0.79)	< 0.001
30‐day readmission^a^	1.02 (1.00–1.05)	0.043
Care fragmentation^c^	3.75 (3.58–3.92)	< 0.001
Failure‐to‐rescue^d^	0.93 (0.89–0.97)	< 0.001
In‐hospital mortality^a^	1.13 (1.09–1.18)	< 0.001

*Note:* Models were adjusted for age, sex, insurance status, ZIP income quartile, weekend admission, operative urgency, surgery type, Elixhauser comorbidity burden, hospital bed size, hospital ownership, hospital teaching status, metropolitan hospital location, and hospital operative volume. Standard errors were clustered at the hospital level.

Abbreviations: CI, confidence interval; IHTR, interhospital transfer; IRR, incidence rate ratio; LOS, length of stay; NTR, non‐transfer; OR, odds ratio; ZIP, Zone Improvement Plan.

^a^ Analytic cohort included the full cohort: total *n* = 1,831,450; NTR *n* = 1,753,952; IHTR *n* = 77,498.

^b^ Index LOS was modeled using count regression and reported as an incidence rate ratio.

^c^ Analytic cohort for care fragmentation included patients with 30‐day readmission: total *n* = 224,054; NTR *n* = 211,311; IHTR *n* = 12,743.

^d^ Analytic cohort for failure‐to‐rescue included patients with a documented complication: total *n* = 627,828; NTR *n* = 578,518; IHTR *n* = 49,310.

IHTR was only modestly associated with 30‐day readmission in the full cohort (aOR, 1.02; 95% CI, 1.00–1.05; *p* = 0.043). In contrast, among readmitted patients, IHTR was strongly associated with care fragmentation (aOR, 3.75; 95% CI, 3.58–3.92; *p* < 0.001). Among patients with complications, IHTR was associated with slightly lower adjusted odds of failure‐to‐rescue (aOR, 0.93; 95% CI, 0.89–0.97; *p* < 0.001), while IHTR was associated with higher adjusted odds of in‐hospital mortality in the full cohort (aOR, 1.13; 95% CI, 1.09–1.18; *p* < 0.001) (Table 3).

### 3.4 Operative Urgency‐Stratified Analyses

The association between IHTR and care fragmentation was consistent after stratification by operative urgency. Among nonelective patients with 30‐day readmission, IHTR was associated with higher odds of care fragmentation (aOR, 4.05; 95% CI, 3.86–4.26; *p* < 0.001). Similarly, among elective patients with 30‐day readmission, IHTR remained associated with care fragmentation (aOR, 3.46; 95% CI, 3.10–3.86; *p* < 0.001) (Table 4).

**TABLE 4:** Stratified association of interhospital transfer with care fragmentation by operative urgency.

Outcome	Adjusted OR (95% CI)	*p* value
Care fragmentation among nonelective patients^a^	4.05 (3.86–4.26)	< 0.001
Care fragmentation among elective patients^b^	3.46 (3.10–3.86)	< 0.001

*Note:* Models were adjusted for age, sex, insurance status, ZIP income quartile, weekend admission, surgery type, Elixhauser comorbidity burden, hospital bed size, hospital ownership, hospital teaching status, metropolitan hospital location, and hospital operative volume. Standard errors were clustered at the hospital level.

Abbreviations: CI, confidence interval; IHTR, interhospital transfer; NTR, non‐transfer; OR, odds ratio; ZIP, Zone Improvement Plan.

^a^ Analytic cohort included patients with 30‐day readmission after nonelective surgery: total *n* = 113,938; NTR *n* = 102,794; IHTR *n* = 11,144.

^b^ Analytic cohort included patients with 30‐day readmission after elective surgery: total *n* = 110,116; NTR *n* = 108,517; IHTR *n* = 1599.

In hospital‐level analyses, greater hospital transfer burden was associated with higher odds of care fragmentation among all readmitted patients (OR, 1.08; 95% CI, 1.08–1.09) and among NTR readmitted patients only (OR, 1.08; 95% CI, 1.07–1.09) (Supporting Information S1: Table 2). Hospital‐level linear rate sensitivity analyses were consistent across hospitals with at least 10, 20, and 30 readmissions (Supporting Information S1: Table 3).

In sensitivity analyses, the association between IHTR and care fragmentation was not materially attenuated after adjustment for resident status, restriction to in‐state residents, or additional adjustment for index‐admission complexity markers (Supporting Information S1: Tables 4 and 5). Fragmented readmissions had higher readmission‐course mortality, discharge against medical advice, readmission cost, and sepsis/renal‐failure markers than non‐fragmented readmissions (Supporting Information S1: Table 6). Procedure‐specific and temporal analyses demonstrated that care fragmentation among readmitted IHTR patients was common across procedures and stable across study years (Supporting Information S1: Tables 7 and 8). Propensity‐based sensitivity analyses, including inverse probability weighting and 1:1 propensity‐score matching, yielded consistent results (Supporting Information S1: Tables 9–12). Sex‐based analyses demonstrated similar IHTR‐care fragmentation associations among male and female patients (Supporting Information S1: Table 13).

## 4 Discussion

Continuity of care after a major surgical procedure is important, particularly when patients require acute care after discharge [21, 23]. Ideally, postoperative concerns are evaluated at the index operative hospital, where surgical teams, operative details, and the patient's postoperative trajectory are known. The current study characterized the association of interhospital transfer (IHTR) with post‐discharge care fragmentation after major surgery and evaluated whether hospital transfer burden was associated with hospital‐level fragmentation. Several findings should be highlighted. First, IHTR was only modestly associated with 30‐day readmission, yet was strongly associated with care fragmentation among readmitted patients. Second, approximately one‐half of readmitted IHTR patients were readmitted to a non‐index hospital, compared with approximately one‐fifth of readmitted non‐transferred patients. Third, greater hospital transfer burden was associated with hospital‐level care fragmentation, including among patients who were not transferred into the index hospitalization.

In the current study, 4.2% of patients were transferred into the index surgical hospitalization, a proportion consistent with prior national estimates of IHTR among surgical populations [1, 2, 6]. Transferred patients differed substantially from non‐transferred patients in comorbidity burden, operative urgency, index length of stay, and discharge disposition. These findings align with prior work characterizing transferred surgical patients as a higher‐acuity population requiring specialized resources [5, 6, 24]. Importantly, the central finding was not simply that transferred patients were readmitted more often; the adjusted association between IHTR and 30‐day readmission was modest. Rather, among patients who were readmitted, IHTR was associated with nearly four‐fold higher odds of care fragmentation. Prior work has similarly identified non‐index readmission as an important marker of postoperative discontinuity [7, 8, 12]. In a systematic review and meta‐analysis, non‐index readmissions accounted for 10%–47% of 30‐day surgical readmissions, and most included studies reported higher mortality among patients with fragmented care [8]. The current findings extend this by demonstrating that transfer into the index surgical hospitalization was strongly associated with the subsequent location of readmission after major surgery. Of note, despite higher complication rates among IHTR patients, adjusted failure‐to‐rescue was slightly lower, a finding that may reflect differences in hospital capability, case selection, or residual differences in complication type and severity among patients treated at transfer‐receiving hospitals [15, 25].

Care fragmentation occurred among approximately one‐half of readmitted IHTR patients in both nonelective and elective surgical cohorts, suggesting that this pattern was not limited to emergency transfer pathways. Adjustment for resident status, restriction to in‐state residents, additional adjustment for index‐admission complexity, and propensity‐based sensitivity analyses did not materially attenuate the association. Sex‐based analyses also demonstrated similar associations among male and female patients. Together, these findings suggest that measured complexity, out‐of‐state residence, operative urgency, and sex did not fully account for the observed association.

The hospital‐level findings further support a systems‐level interpretation. Greater hospital transfer burden was associated with higher care fragmentation among all readmitted patients and among non‐transferred readmitted patients. This finding suggests that transfer‐heavy hospitals may serve broader geographic catchment areas and referral networks that shape post‐discharge care patterns across the surgical population. These findings should not be interpreted as evidence that transfer‐heavy hospitals produce care fragmentation; rather, transfer burden may function as a marker of regional referral role and geographic reach [18, 19].

These findings have implications for how postoperative readmissions are interpreted within regionalized surgical systems. The central issue identified in the current study was a marked difference in where readmissions occurred. Readmission to a non‐index hospital is not necessarily inappropriate, particularly when patients live far from the operative center or require urgent local evaluation. However, non‐index readmission indicates that post‐discharge acute care is occurring outside the hospital where the index operation was performed, making subsequent care more dependent on interfacility communication, availability of operative and discharge information, and clear escalation pathways. From a quality‐measurement perspective, readmission metrics that focus only on whether a patient is readmitted may miss this dimension of postoperative continuity. Similarly, length‐of‐stay and failure‐to‐rescue metrics may require contextual interpretation at referral centers if transfer burden is not considered, as transferred patients represent a higher‐acuity referral population. Incorporating transfer status, hospital transfer burden, or non‐index readmission rates into quality dashboards may help distinguish readmission frequency from readmission continuity. At the same time, transfer‐based adjustment should be applied cautiously, as fragmented readmission may reflect both unavoidable geography and potentially modifiable gaps in discharge communication, referral‐hospital handoff, and early post‐discharge access. Transfer burden may therefore be most useful as a stratification or contextual reporting measure rather than as a blanket exemption from performance accountability [26, 27].

This study should be interpreted in light of several limitations. The NRD lacks physiologic data, operative details, laboratory values, and granular clinical information; therefore, comorbidities, complications, and readmission condition markers were defined using administrative coding. Although NRD hospital identifiers allowed identification of same‐hospital versus different‐hospital readmissions, the database does not identify the specific nonindex hospital or whether patients returned to the original referring facility, a local hospital closer to home, or another referral center. The NRD also lacks patient‐to‐hospital distance, travel time, local hospital availability, referral‐region information, shared electronic health record status, health information exchange participation, and discharge communication quality. The NRD captures same‐state readmissions only and does not capture out‐of‐state readmissions.14 Finally, residual differences in transfer indication, including escalation for higher‐acuity care versus planned referral for complex surgery, as well as readmission acuity, readmission indication, and patient preference could not be fully accounted for.

In conclusion, IHTR was strongly associated with care fragmentation after major surgery, with only a modest association with 30‐day readmission overall. Greater hospital transfer burden was also associated with hospital‐level care fragmentation, including among patients who were not transferred into the index hospitalization. These findings suggest that transfer status and hospital transfer burden may identify patients and hospitals at increased risk for fragmented post‐discharge acute care within regionalized surgical systems. Strategies to improve postoperative continuity should prioritize structured discharge communication, direct contact pathways, and early follow‐up between index surgical hospitals and local acute‐care facilities.

## Author Contributions

**Rida Ejaz:** conceptualization, investigation, methodology, writing – review and editing, writing – original draft, formal analysis, data curation. **Qaidar Alizai:** conceptualization, writing – review and editing, writing – original draft, validation. **Lorenza Arena:** writing – review and editing, methodology, validation, writing – original draft, conceptualization. **Kizuki Yuza:** conceptualization, writing – original draft, writing – review and editing, visualization. **Charalampos M. Charalampous:** conceptualization, writing – review and editing, visualization. **Abdulaziz Elemosho:** visualization, writing – review and editing, conceptualization. **Timothy M. Pawlik:** writing – review and editing, conceptualization, supervision, project administration, visualization.

## Funding

The authors have nothing to report.

## Disclosures

The authors have nothing to report.

## Conflicts of Interest

The authors declare no conflicts of interest.

## Supporting information


Supporting Information S1

**Figure S1:** Study definition by Transfer and Readmission Pathways.

## Data Availability

The data that support the findings of this study are available from Healthcare Cost and Utilization Project (HCUP), AHRQ. Restrictions apply to the availability of these data, which were used under license for this study. Data are available from https://hcup‐us.ahrq.gov/nrdoverview.jsp with the permission of Healthcare Cost and Utilization Project (HCUP), AHRQ.
