# Supplementary material for: The Unfinished Journey After Major Surgery: Interhospital Transfer and Care Fragmentation
Source: World J Surg. 2026 Jul 14;50(9):2565–73. doi: 10.1002/wjs.70502 (PMC13576586; doi:10.1002/wjs.70502)
Supplement: Supplementary file 1 [file WJS-50-2565-s002.docx]

Supplementary Table 1. Missingness assessment

| **Variable group** | **Missingness** | **Handling** |
| --- | --- | --- |
| Primary exposure and main covariates | 0% | No missing-category indicator required |
| Care fragmentation | Structurally undefined among patients without 30-day readmission | Evaluated only among patients with 30-day readmission |
| Care fragmentation among readmitted patients | 0/224,054 missing | Included in readmitted cohort analyses |
| Care fragmentation was defined only among patients with 30-day readmission; therefore, patients without 30-day readmission were not considered missing for this outcome but were outside the analytic denominator. Missingness was assessed for the primary exposure and all covariates included in the main adjusted models. No missingness was identified for the primary exposure or covariates included in the main models. IHTR, interhospital transfer; NTR, non-transfer. | | |

Supplementary Table 2. Hospital-level association between transfer burden and care fragmentation

| **Hospital-level analysis** | **Hospitals included** | **Model** | **aOR, 95% CI** | **p value** |  |
| --- | --- | --- | --- | --- | --- |
| All readmitted patients | 8,227 | Binomial GLM | 1.08 (1.08–1.09) | <0.001 |  |
| Non-transferred readmitted patients only | 8,054 | Binomial GLM | 1.08 (1.07–1.09) | <0.001 |  |
| Hospital transfer burden was defined as the proportion of major surgical patients at each index hospital admitted via IHTR. Hospital-level binomial generalized linear models used the number of fragmented readmissions as the numerator and total 30-day readmissions as the denominator. For the non-transferred-only analysis, the numerator was fragmented readmissions among NTR patients and the denominator was total 30-day readmissions among NTR patients. Models were adjusted for log-transformed hospital operative volume, hospital bed size, hospital ownership, hospital teaching status, and metropolitan hospital location. No minimum readmission-volume threshold was used in the primary hospital-level models. CI, confidence interval; GLM, generalized linear model; IHTR, interhospital transfer; NTR, non-transfer; OR, odds ratio. | | | | | |

Supplementary Table 3. Hospital-level linear rate sensitivity analyses

| **Sensitivity analysis** | **Hospitals included** | **β per 1-percentage-point higher transfer burden** | **95% CI** | **p value** |
| --- | --- | --- | --- | --- |
| ≥10 readmissions | 4,314 | 1.37 | 1.19–1.56 | <0.001 |
| ≥20 readmissions | 3,066 | 1.72 | 1.61–1.84 | <0.001 |
| ≥30 readmissions | 2,367 | 1.80 | 1.68–1.93 | <0.001 |
| ≥10 non-transfer readmissions | 4,269 | 1.26 | 1.14–1.38 | <0.001 |
| ≥20 non-transfer readmissions | 3,009 | 1.44 | 1.31–1.56 | <0.001 |
| ≥30 non-transfer readmissions | 2,287 | 1.54 | 1.42–1.67 | <0.001 |
| Linear rate models were performed as sensitivity analyses to evaluate the stability of hospital-level findings after excluding hospitals with sparse readmission denominators. The primary hospital-level analysis used binomial generalized linear models and did not require a minimum readmission-volume threshold. β estimates represent the absolute percentage-point difference in hospital-level care fragmentation rate per 1-percentage-point higher hospital transfer burden. Separate sensitivity analyses were performed among all readmitted patients and among NTR readmitted patients only. CI, confidence interval; IHTR, interhospital transfer; NTR, non-transfer. | | | | |

Supplementary Table 4. Resident-state sensitivity analyses for care fragmentation

| **Model** | **Population** | **aOR, 95% CI** | **p value** |  |
| --- | --- | --- | --- | --- |
| Primary model | All readmitted patients | 3.75 (3.58–3.92) | <0.001 |  |
| Adjusted for resident status | All readmitted patients | 3.67 (3.51–3.84) | <0.001 |  |
| Restricted to in-state residents | RESIDENT=1 | 3.62 (3.46–3.79) | <0.001 |  |
| Care fragmentation was evaluated among patients with 30-day readmission. Models adjusted for age, sex, insurance status, ZIP income quartile, weekend admission, operative urgency, surgery type, Elixhauser comorbidity burden, hospital bed size, hospital ownership, hospital teaching status, metropolitan hospital location, and hospital operative volume. The resident-status model additionally adjusted for whether the patient’s state of residence matched the hospital state. Standard errors were clustered at the hospital level. CI, confidence interval; IHTR, interhospital transfer; OR, odds ratio; ZIP, Zone Improvement Plan. | | | | |

Supplementary Table 5. Index-complexity sensitivity analyses for care fragmentation

| **Model** | **Additional adjustment** | **aOR, 95% CI** | **p value** |  |
| --- | --- | --- | --- | --- |
| Primary model | Baseline covariates | 3.75 (3.58–3.92) | <0.001 |  |
| Sensitivity model 1 | Any complication, index LOS, discharge home | 3.73 (3.57–3.91) | <0.001 |  |
| Sensitivity model 2 | Any complication, LOS >75th percentile, discharge home | 3.74 (3.57–3.91) | <0.001 |  |
| Care fragmentation was evaluated among patients with 30-day readmission. The primary model adjusted for age, sex, insurance status, ZIP income quartile, weekend admission, operative urgency, surgery type, Elixhauser comorbidity burden, hospital bed size, hospital ownership, hospital teaching status, metropolitan hospital location, and hospital operative volume. Sensitivity models additionally adjusted for markers of index-admission complexity. These variables were not included in the primary model because they occurred during the index hospitalization and may be on the clinical pathway after IHTR. Standard errors were clustered at the hospital level. CI, confidence interval; IHTR, interhospital transfer; LOS, length of stay; OR, odds ratio; ZIP, Zone Improvement Plan. | | | | |

Supplementary Table 6. Readmission-course outcomes by readmission destination

| **Outcome** | **Non-fragmented readmission** | **Fragmented readmission** | **p value** |
| --- | --- | --- | --- |
| Readmission mortality | 3.6% | 5.6% | <0.001 |
| Readmission LOS, median, days | 5 | 5 |  |
| Readmission LOS, mean, days | 7.48 | 7.98 |  |
| Readmission cost, median | $11,469 | $12,240 |  |
| Discharge against medical advice | 0.6% | 1.5% | <0.001 |
| Sepsis/renal-failure marker | 39.5% | 49.3% | <0.001 |
| Analyses were restricted to patients with 30-day readmission. Fragmented readmission was defined as readmission to a hospital different from the index surgical hospital. These analyses were interpreted descriptively because residual differences in readmission acuity, indication for readmission, patient geography, and patient preference could not be fully captured. Continuous variables are presented as median or mean as specified. LOS, length of stay. | | | |

Supplementary Table 7. Procedure-specific care fragmentation among patients with 30-day readmission

| **Procedure** | **Readmitted patients, N** | **Care fragmentation among all readmitted patients** | **Care fragmentation among IHTR readmitted patients** |
| --- | --- | --- | --- |
| AAA | 30,144 | 9,223 (30.6) | 1,018/1,619 (62.9) |
| Esophagectomy | 2,645 | 483 (18.3) | 41/89 (46.1) |
| Hepatectomy | 3,761 | 659 (17.5) | 24/89 (27.0) |
| Rectal resection | 19,316 | 3,053 (15.8) | 219/442 (49.6) |
| CABG | 56,700 | 13,822 (24.4) | 3,808/7,431 (51.2) |
| Colectomy | 87,379 | 15,127 (17.3) | 1,054/2,389 (44.1) |
| Pancreatectomy | 8,835 | 1,126 (12.7) | 95/267 (35.6) |
| Pneumonectomy | 15,274 | 3,258 (21.3) | 165/417 (39.6) |
| Analyses were restricted to patients with 30-day readmission. Care fragmentation was defined as readmission to a hospital different from the index surgical hospital. Percentages in the third column were calculated among all readmitted patients within each procedure group. Percentages in the fourth column were calculated among readmitted IHTR patients within each procedure group. AAA, abdominal aortic aneurysm; CABG, coronary artery bypass grafting; IHTR, interhospital transfer. | | | |

Supplementary Table 8. Temporal trends and year-stratified sensitivity analyses

| **Year** | **Care fragmentation among IHTR readmitted patients** | **aOR, 95% CI** | **p value** |  |
| --- | --- | --- | --- | --- |
| 2016 | 51.5% | 3.73 (3.37–4.12) | <0.001 |  |
| 2017 | 51.7% | 3.83 (3.48–4.22) | <0.001 |  |
| 2018 | 49.3% | 3.66 (3.31–4.04) | <0.001 |  |
| 2019 | 48.9% | 3.61 (3.25–4.00) | <0.001 |  |
| 2020 | 50.8% | 3.93 (3.54–4.35) | <0.001 |  |
| Care fragmentation was evaluated among patients with 30-day readmission. Year-stratified models adjusted for age, sex, insurance status, ZIP income quartile, weekend admission, operative urgency, surgery type, Elixhauser comorbidity burden, hospital bed size, hospital ownership, hospital teaching status, metropolitan hospital location, and hospital operative volume. Standard errors were clustered at the hospital level. In the model additionally adjusted for calendar year, IHTR remained associated with care fragmentation: adjusted OR, 3.74; 95% CI, 3.57–3.91. CI, confidence interval; IHTR, interhospital transfer; OR, odds ratio; ZIP, Zone Improvement Plan. | | | | |

Supplementary Table 9. Propensity-based sensitivity analyses for care fragmentation

| **Analysis** | **Adjusted OR/OR** **95% CI** | **p value** |
| --- | --- | --- |
| Primary multivariable model | aOR 3.75 (3.58–3.92) | <0.001 |
| Truncated IPTW model | OR 3.96 (3.76–4.19) | <0.001 |
| Doubly adjusted truncated IPTW model | aOR 3.78 (3.58–3.99) | <0.001 |
| 1:1 propensity-score matched model | OR 4.02 (3.76–4.30) | <0.001 |
| Care fragmentation was evaluated among patients with 30-day readmission. The primary multivariable model adjusted for age, sex, insurance status, ZIP income quartile, weekend admission, operative urgency, surgery type, Elixhauser comorbidity burden, hospital bed size, hospital ownership, hospital teaching status, metropolitan hospital location, and hospital operative volume. IPTW models used stabilized inverse probability of treatment weights truncated at the 1st and 99th percentiles. Robust standard errors were clustered at the hospital level. CI, confidence interval; IPTW, inverse probability of treatment weighting; OR, odds ratio; ZIP, Zone Improvement Plan. | | |

Supplementary Table 10. Propensity-score matching diagnostics

| **Balance diagnostic** | **Before matching** | **After matching** |
| --- | --- | --- |
| Mean standardized bias, % | 24.0 | 1.3 |
| Median standardized bias, % | 10.2 | 1.0 |
| Pseudo R² | 0.178 | 0.001 |
| LR chi-square p value | <0.001 | 0.051 |
| Rubin’s B | 130.2 | 5.9 |
| Rubin’s R | 0.70 | 1.08 |
| Variables with variance ratios outside recommended range, % | 89 | 44 |
| Propensity-score matching used 1:1 nearest-neighbor matching with a caliper of 0.05 and common support restriction. The propensity model included age, sex, insurance status, ZIP income quartile, weekend admission, operative urgency, surgery type, Elixhauser comorbidity burden, hospital bed size, hospital ownership, hospital teaching status, metropolitan hospital location, and hospital operative volume. LR, likelihood ratio; ZIP, Zone Improvement Plan. | | |

Supplementary Table 11. Care fragmentation in the 1:1 propensity-score matched cohort

| **Outcome** | **Non-transfer patients** | **IHTR patients** | **OR/PP (95% CI)** |
| --- | --- | --- | --- |
| Odds of care fragmentation | Reference | — | 4.02 (3.76–4.30) |
| Care fragmentation, % | 20.2 | 50.4 | 30.2pp (29.0–31.4) |
| Care fragmentation was evaluated among matched patients with 30-day readmission. The absolute difference was derived from the matched comparison; the odds ratio was estimated using the matched cohort with robust standard errors clustered at the hospital level. CI, confidence interval; IHTR, interhospital transfer; OR, odds ratio. | | | |

Supplementary Table 12. Propensity-score matched cohort construction

| **Cohort characteristic** | **Value** |
| --- | --- |
| Total patients with 30-day readmission | 224,054 |
| IHTR patients eligible for matching | 12,743 |
| IHTR patients successfully matched | 12,742 |
| IHTR patients outside common support | 1 |
| Non-transfer patients on common support | 211,311 |
| Matched analytic sample for weighted matched model | 23,488 |
| Matching method | 1:1 nearest-neighbor matching |
| Caliper | 0.05 |
| Common support restriction | Yes |
| Analyses were restricted to patients with 30-day readmission. IHTR, interhospital transfer. | |

Supplementary Table 13. Sex-based analyses of interhospital transfer and care fragmentation

| **Analysis** | **Analytic cohort** | **Outcome** | **aOR, 95% CI** | **p value** |
| --- | --- | --- | --- | --- |
| Female vs male patients | Full surgical cohort, N=1,831,450 | Interhospital transfer | 0.98 (0.96–1.00) | 0.041 |
| Male patients | Readmitted patients, n=125,150 | Care fragmentation associated with IHTR | 3.77 (3.57–3.99) | <0.001 |
| Female patients | Readmitted patients, n=98,904 | Care fragmentation associated with IHTR | 3.64 (3.41–3.89) | <0.001 |
| Models adjusted for age, insurance status, ZIP income quartile, weekend admission, operative urgency, surgery type, Elixhauser comorbidity burden, hospital bed size, hospital ownership, hospital teaching status, metropolitan hospital location, and hospital operative volume. The full-cohort model evaluating female versus male patients additionally included sex as the primary independent variable. Sex-stratified care-fragmentation models excluded sex from the covariate set. Standard errors were clustered at the hospital level. aOR, adjusted odds ratio; CI, confidence interval; IHTR, interhospital transfer; ZIP, Zone Improvement Plan. | | | | |

Supplementary Figure 1: Study definition by Transfer and Readmission Pathways

Interhospital transfer was defined as transfer into the index operative hospitalization. Care fragmentation was evaluated only among patients discharged alive after the index operation who subsequently experienced a 30-day readmission. Non-fragmented readmission was defined as readmission to the index surgical hospital, whereas fragmented readmission was defined as readmission to a hospital different from the index surgical hospital. Transfer out during the index hospitalization was not classified as care fragmentation unless followed by a qualifying 30-day readmission. The NRD cannot determine whether the initial transfer reflected escalation or rescue care, management of a complication at the referring hospital, elective referral to a high-complexity center, or de-escalation/readmission closer to home.
